# IGF-1 as a Biomarker for Symptom Severity in Adult Traumatic Brain Injury: Evidence from an Observational Study

**DOI:** 10.1089/neur.2025.0009

**Published:** 2025-04-21

**Authors:** Justin Weppner, Kimberly Rosenthal, Jennifer Bath, Tonja Locklear, Melissa Martinez

**Affiliations:** ^1^ Department of Internal Medicine, Virginia Tech Carilion School of Medicine, Roanoke, Virginia, USA.; ^2^ Department of Internal Medicine, Carilion Clinic, Roanoke, Virginia, USA.; ^3^ Department of Internal Medicine, Edward Via College of Osteopathic Medicine, Blacksburg, Virginia, USA.; ^4^ Department of Physical Medicine and Rehabilitation, Virginia Health Sciences at Old Dominion University, Norfolk, Virginia, USA.; ^5^ Department of Surgery, Carilion Clinic, Roanoke, Virginia, USA.

**Keywords:** biomarker, growth hormone deficiency, insulin-like growth factor 1, neuroendocrine dysfunction, traumatic brain injury

## Abstract

Traumatic brain injury (TBI)-related growth hormone deficiency is often undertreated, despite documented physical, metabolic, and neuropsychiatric effects. Insulin-like growth factor (IGF-1), with neuroreceptors located in brain regions responsible for learning, memory, and mood, regulates cerebral blood flow, neurogenesis, and neuroplasticity. The aim of this study was to determine associations between IGF-1 levels and post-TBI symptom severity, anxiety, and depression. This retrospective observational study at an Academic Brain Injury Center included participants evaluated 3–12 months post-TBI with available IGF-1 values and complete Rivermead Post-Concussion Symptoms Questionnaire (RPQ-13), Generalized Anxiety Disorder-7 (GAD-7), and Patient Health Questionnaire-9 (PHQ-9) responses. Patients under 18 or over 65 and those with incomplete data were excluded. Participants were grouped by TBI severity: mild (Glasgow Coma Scale [GCS] 13–15) and moderate-to-severe (GCS < 13). IGF-1 *Z*-scores were standardized for age and gender. Significant negative correlations were found between IGF-1 levels and RPQ-13, GAD-7, and PHQ-9 scores across all TBI severity groups, with lower IGF-1 *Z*-scores correlating with higher symptoms of TBI, depression, and anxiety. The Generalized Linear Models showed that the IGF-1 *Z-*score is a significant predictor for GAD-7, PHQ-9, and RPQ-13. Specifically, a one-point increase in the IGF-1 *Z-*score is associated with a 29.85% decrease in anxiety symptoms on the GAD-7, a 16.30% reduction in depression severity on the PHQ-9, and a 39.23% decrease in post-TBI symptom severity on the RPQ-13. Findings suggest that decreased IGF-1 is associated with increased post-injury symptom severity, depression, and anxiety. Future studies should explore IGF-1 as a biomarker for TBI symptom severity.

## Introduction

Traumatic brain injury (TBI) is a disruption of normal brain function due to an external mechanical force causing neuron dysfunction and death.^1–3^ Hypopituitarism is a common and frequently underdiagnosed complication of TBI.^4^ Specifically, growth hormone deficiency (GHD) has known, significant deleterious physical, metabolic, and neuropsychological effects post-injury.^4–6^ Though GHD is the most common form of pituitary deficiency in chronic TBI, with reported prevalence of 3–34%, it is often undertreated.^2,7^ This is, in part, due to the nonspecific symptomatology of GHD, which includes fatigue, depression, sleep disturbance, and memory impairment.^4^

Insulin-like growth factor (IGF-1) functions as the major mediator of growth hormone (GH) activity. IGF-1 enters the brain from circulation to regulate cerebral blood flow and promote neurogenesis and neuroplasticity.^8–10^ IGF-1 neuroreceptors are preferentially expressed in the prefrontal cortex, hippocampus, parahippocampal areas, and amygdala. These regions are involved in learning, memory, and mood.^9,11–13^

The cerebral cortex, hippocampus, hypothalamus, and pituitary gland are often damaged in TBI and region-specific changes in IGF-1 expression as well as decreased serum IGF-1 level have been reported in both acute and chronic TBI.^1,3,6,9^ This disruption may be exacerbated by secondary cascades involving inflammatory responses and oxidative stress, which further impair IGF-1 signaling pathways, potentially leading to deficits in neuroprotection and repair mechanisms.^8–10^ Review of the literature reveals limited studies exploring potential associations between IGF-1 levels and post-TBI symptom severity and mood disorders in humans, though data from animal studies are compelling. Animal models have demonstrated associations between circulating IGF-1 level and brain injury-induced cognitive dysfunction and anxiety and suggest a neuroprotective role for IGF-1 in brain injury.^3,6^

We aimed to evaluate whether associations exist between IGF-1 levels and (1) post-injury symptom severity as measured by the Rivermead Post-Concussion Symptoms Questionnaire (RPQ-13), (2) post-injury depression on the Patient Health Questionnaire-9 (PHQ-9), and (3) anxiety as measured by the Generalized Anxiety Disorder-7 (GAD-7) following TBI. We hypothesized lower IGF-1 level to be associated with increased symptom severity and incidence of depression and anxiety after TBI.

## Materials and Methods

### Study protocol

This retrospective observational study was conducted at an Academic Brain Injury Center from September 1, 2021, to September 1, 2023. Participants included were those evaluated at the Brain Injury Center within 3–12 months of their TBI, had IGF-1 values available, and complete RPQ-13, GAD-7, and PHQ-9 responses.^14–20^ Exclusion criteria were patients younger than 18 or older than 65 years, incomplete RPQ-13, PHQ-9, or GAD-7 questionnaires, and IGF-1 data unavailability. In our clinical practice, patients with symptomatic TBI are routinely screened for pituitary dysfunction, including IGF-1, at 3–6 months and 12 months post-injury. Furthermore, assessments utilizing the GAD-7, PHQ-9, and RPQ-13 are performed at each clinical encounter.^21–23^

Data were extracted from electronic medical records and included RPQ-13, PHQ-9, and GAD-7 scores completed at the visit directly prior to the IGF-1 level blood draw. This study was approved by the Carilion Clinic institutional review board (IRB) committee (IRB-23-2068) prior to data collection. Patients were categorized into two groups based on TBI severity: the mild TBI (mTBI, *n* = 138) group and the moderate-to-severe TBI (msTBI, *n* = 102) group. mTBI included subjects with TBI and Glasgow Coma Scale (GCS) score of 13–15, loss of consciousness lasting less than 30 min, and post-traumatic amnesia duration of less than 24 h. msTBI was defined as having a GCS score of less than 13 after 30 min, loss of consciousness for more than 30 min, or post-traumatic amnesia lasting greater than 24 h. As IGF-1 levels are influenced by age and gender, IGF-1 scores were converted to *Z-*scores to standardize across all age groups. The *Z-*scores were used in all analyses involving IGF-1 throughout the study.

### Statistics

All analyses compared the outcomes between the mTBI and msTBI groups. Descriptive statistical analyses were conducted using the median two-sample test for the numeric fields and Fisher’s exact test for the categorical fields. Median two-sample test was also used to compare the average scores on all three questionnaires between the patients with mTBI and msTBI. To analyze the relationship between the abnormality of the IGF-1 scores and RPQ-13, PHQ-9, GAD-7, and Kendall’s Tau (τ) correlations were used (Supplementary Table S1). This correlation test was a robust option for noncontinuous data. Three independent Generalized Linear Models (GLMs) with a Gamma distribution and a log link function were used to model the three outcome variables GAD 7 score, PHQ-9 score, and RPQ 13. The model accounts for the nonlinear relationship between the predictors (age, body mass index, IGF-1 *Z-*scores, gender, race, and TBI severity) and the three response variables by modeling the logarithm of the expected value of each as a linear function of the explanatory variables. Analyses were conducted using SAS Enterprise Guide 8.3 (SAS, Inc., Cary, NC). The level of statistical significance was established as *α* = 0.05.

## Results

### Demographics

Data indicate that mechanism of injury differed significantly between the mild and msTBI groups (*p* < 0.0001), with the mild group experiencing more blunt head injuries (21.74%) and falls (63.04%) as compared with the msTBI group, which had more motor vehicle collisions (50.98%) and penetrating head injuries (2.94%). Additionally, the mild and msTBI groups presented to the clinic, on average, at 64 and 113 days post-injury, respectively (*p* < 0.0001). A similar trend was observed with time to lab draw, with mean lab draw occurring at 126 days post-injury for the mild TBI group and at 181 days post-injury for the msTBI group (*p* < 0.0001) (Table 1).

**Table 1.** Demographic and Clinical Comparisons

	Mild TBI *N* = 138	Moderate/severe TBI *N* = 102	*p* value
Age (years)	41 (24)	36 (24)	0.0573
BMI (kg/m^2^)	29 (8)	28 (9)	0.2521
Gender			1.0000
Female	56 (40.58%)	42 (41.18%)	
Male	82 (59.42%)	60 (58.82%)	
Race			0.7308
Black	14 (10.14%)	8 (7.84%)	
White	115 (83.33%)	89 (87.25%)	
Other	9 (6.52%)	5 (4.90%)	
Mechanism of injury			<0.0001^*^
Blunt head injury and assaults	30 (21.74%)	16 (15.69%)	
Falls	87 (63.04%)	31 (30.39%)	
Motor vehicle collision	21 (15.22%)	52 (50.98%)	
Penetrating head injury	0 (0.00%)	3 (2.94%)	
Depression history			0.5191
No	112 (81.16%)	79 (77.45%)	
Yes	26 (18.84%)	23 (22.55%)	
Anxiety history			0.6802
No	121 (87.68%)	92 (90.20%)	
Yes	17 (12.32%)	10 (9.80%)	
Time to clinic presentation (days)	64 (54)	113 (127)	<0.0001^*^
Time to lab draw (days)	126 (47)	181 (129)	<0.0001^*^

Data are presented as median (interquartile range) or *n* (%). Analyses were conducted using either median two-sample test or Fisher’s exact test with 5% level of significance.

^*^ Statistically significant

BMI, body mass index; TBI, traumatic brain injury.

### Rivermead Post-Concussion Symptoms Questionnaire, Patient Health Questionnaire-9, Generalized Anxiety Disorder-7

**FIG. 1.**
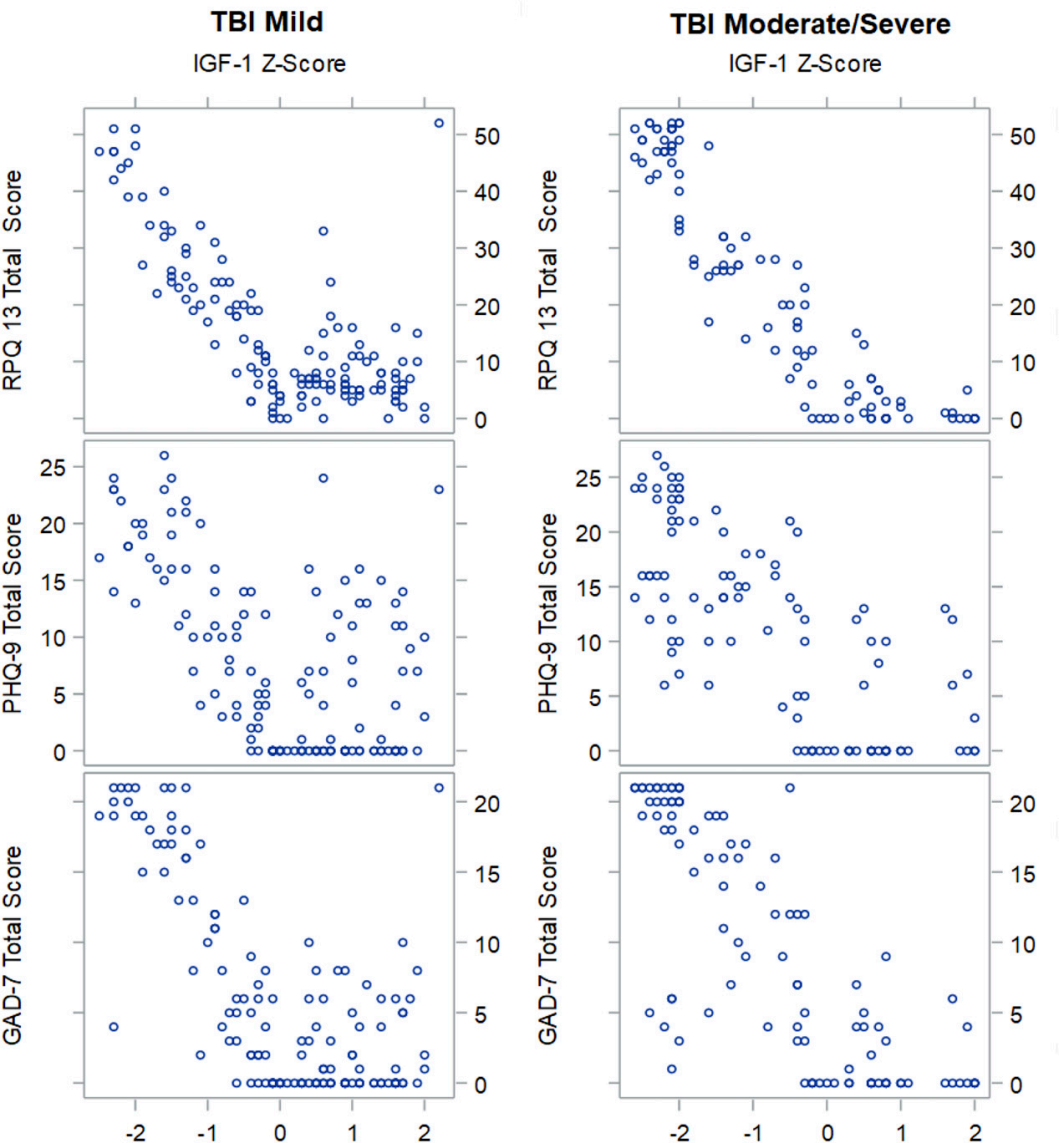
Graphical representation of Kendall’s Tau data demonstrating strong negative correlations between IGF-1 *Z-*scores and RPQ-13, PHQ-9, and GAD-7 scores that reach statistical significance (*p* < 0.001) for mild TBI and moderate-to-severe TBI groups. IGF-1 *Z-*scores are represented on the *x*-axis. RPQ-13, PHQ-9, and GAD-7 scores are represented on the *y*-axis. IGF-1, insulin-like growth factor 1; GAD-7, Generalized Anxiety Disorder 7; PHQ-9, Patient Health Questionnaire 9; RPQ-13, Rivermead Post-Concussion Symptoms Questionnaire 13; TBI, traumatic brain injury.

Median two-sample *t*-tests comparing mTBI and msTBI showed statistically significant increases in RPQ-13, PHQ-9, and GAD-7 scores with increasing TBI severity (*p* < 0.05, Table 2). The results of the Kendall’s Tau correlations show that all data within each level of TBI severity were statistically significant (*p* < 0.001) (Fig. 1). All correlations within each level of TBI severity were negative, indicating that as the IGF-1 *Z-*score increased, RPQ-13, PHQ-9, and GAD-7 scores significantly decreased.

**Table 2.** Outcome Comparisons of RPQ-13, PHQ-9, and GAD-7 Scores Between Mild and Moderate-Severe TBI Groups

	Mild TBI *N* = 138	Moderate/severe TBI *N* = 102	*p* value
RPQ-13 score	10 (17)	21.5 (42)	0.0054
PHQ-9 score	5.5 (14)	12 (18)	0.0030
GAD-7 score	3 (10)	7 (19)	0.0151

Data are presented as median (interquartile range) and analyses were conducted using median two-sample test with 5% level of significance.

GAD-7, Generalized Anxiety Disorder 7; PHQ-9, Patient Health Questionnaire 9; RPQ-13, Rivermead Post-Concussion Symptoms Questionnaire 13; TBI, traumatic brain injury.

Analysis revealed statistically significant (*p* < 0.001), negative Kendall’s Tau correlations between IGF-1 *Z-*scores and RPQ-13, GAD-7, and PHQ-9 scores for mTBI and msTBI groups. Specifically, moderate, negative correlations within the mTBI group between IGF-1 *Z-*scores and PHQ-9 and GAD-7 scores ( τ =−0.39, *p* < 0.001; τ = −0.47, *p* < 0.001, respectively) as well as a strong, negative correlation between IGF-1 *Z-*scores and RPQ-13 scores (τ = −0.51, *p* < 0.001) (Table 3) were observed. Additionally, there was a very strong, negative correlation within the msTBI group between IGF-1 *Z-*scores and RPQ-13 scores ( τ=−0.76, *p* < 0.001) as well as strong, negative correlations between IGF-1 *Z-*scores and PHQ-9, and GAD-7 scores (τ = −0.56 p < 0.001; τ = −0.66 p < 0.001) (Table 3).

**Table 3.** Kendall’s Tau Correlations for IGF-1 Z-Scores and RPQ-13, PHQ-9, and GAD-7 Scores for Mild TBI and Moderate-Severe TBI Groups

	IGF-1 *Z-*score	
	Mild TBI	Moderate/severe TBI
RPQ-13 total score	−0.51	−0.76
PHQ-9 total score	−0.39	−0.56
GAD-7 total score	−0.47	−0.66

GAD-7, Generalized Anxiety Disorder 7; IGF-1, insulin-like growth factor-1; PHQ-9, Patient Health Questionnaire 9; RPQ-13, Rivermead Post-Concussion Symptoms Questionnaire 13; TBI, traumatic brain injury.

### Pre-morbid anxiety and depression

**FIG. 2.**
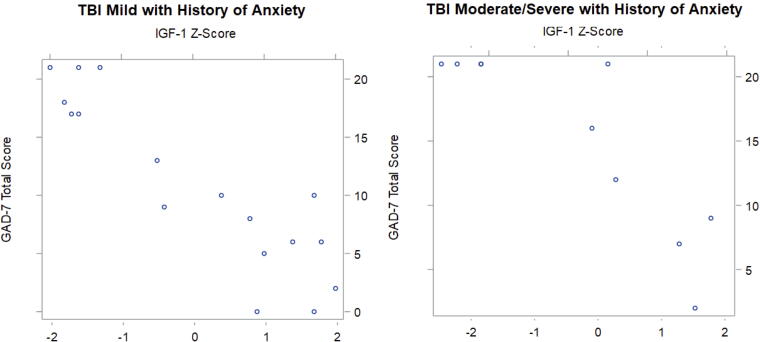
Graphical representation of Kendall’s Tau data demonstrating strong negative correlations between IGF-1 *Z-*scores and GAD-7 scores that reach statistical significance (*p* < 0.001) for mild TBI and moderate-to-severe TBI groups with a history of anxiety. IGF-1 *Z-*scores are represented on the *x*-axis and GAD-7 scores are represented on the *y*-axis. IGF-1, insulin-like growth factor 1; GAD-7, Generalized Anxiety Disorder 7; TBI, traumatic brain injury.

GAD-7 and PHQ-9 data were further divided into those with and without pre-morbid anxiety or depression, respectively. For patients with mTBI and preinjury anxiety, a strong, negative correlation between IGF-1 *Z-*scores and GAD-7 scores that reached statistical significance was observed ( τ = −0.68, *p* = 0.0002) (Table 4, Fig. 2). For the msTBI group with pre-morbid anxiety, there was a very strong, negative correlation between IGF-1 *Z-*score and GAD-7 scores that reached statistical significance (τ = −0.74, *p* = 0.0052). Kendall’s Tau scores comparing IGF-1 *Z-*scores with GAD-7 scores for mTBI and msTBI groups without pre-morbid anxiety were less negative than their counterparts with anxiety, though correlations were still strong and statistically significant (mTBI: τ = −0.49, p < 0.0001; msTBI: τ = −0.67, p < 0.0001).

**Table 4.** Kendall’s Tau Correlations Between IGF-1 Z-Scores and GAD-7 Scores for Mild TBI and Moderate-Severe TBI With and Without Pre-Morbid Anxiety

GAD-7 score	IGF-1 *Z-*score			
	History of anxiety		No history of anxiety	
	Mild TBI *N* = 17	Moderate/severe TBI *N* = 10	Mild TBI *N* = 121	Moderate/severe TBI *N* = 92
	−0.68 (*p* = 0.0002)	−0.74 (*p* = 0.0052)	−0.49 (*p* < 0.0001)	−0.67 (*p* < 0.0001)

GAD-7, Generalized Anxiety Disorder 7; IGF-1, insulin-like growth factor-1; TBI, traumatic brain injury.

**FIG. 3.**
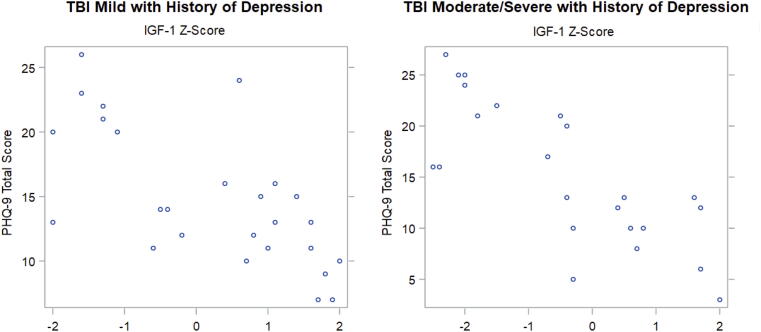
Graphical representation of Kendall’s Tau data demonstrating strong negative correlations between IGF-1 *Z-*scores and PHQ-9 scores that reach statistical significance (*p* < 0.001) for mild TBI and moderate-to-severe TBI groups with a history of depression. IGF-1 *Z-*scores are represented on the *x*-axis and PHQ-9 scores are represented on the *y*-axis. IGF-1, insulin-like growth factor 1; PHQ-9, Patient Health Questionnaire 9; TBI, traumatic brain injury.

For patients with mTBI and pre-morbid depression, a strong, negative correlation between IGF-1 *Z-*scores and PHQ-9 scores that reached statistical significance was seen (τ = −0.51, *p* = 0.0004) (Table 5, Fig. 3). For the msTBI group with pre-morbid depression, a similarly strong, negative and statistically significant correlation between IGF-1 *Z-*score and PHQ-9 scores was observed (τ = −0.63 p < 0.0001) Kendall’s Tau scores comparing IGF-1 *Z-*scores with PHQ-9 scores for mTBI and msTBI groups without preinjury depression were similar to their counterparts with a history of depression (mTBI: τ = −0.52, p < 0.0001; msTBI: τ = −0.63, p < 0.0001).

**Table 5.** Kendall’s Tau Correlations Between IGF-1 Z-Scores and PHQ-9 Scores for Mild TBI and Moderate-Severe TBI Patients With and Without Pre-Morbid Depression

PHQ-9 score	IGF-1 *Z-*score			
	History of depression		No history of depression	
	Mild TBI *N* = 26	Moderate/severe TBI *N* = 23	Mild TBI *N* = 112	Moderate/severe TBI *N* = 79
	−0.51 (*p* = 0.0004)	−0.63 (*p* < 0.0001)	−0.52 (*p* < 0.0001)	−0.63 (*p* < 0.0001)

IGF-1, insulin-like growth factor-1; PHQ-9, Patient Health Questionnaire 9; TBI, traumatic brain injury.

### Generalized Anxiety Disorder-7 Generalized Linear Model

IGF-1 *Z-*score is the only statistically significant predictor of the GAD 7 score (*β* = −0.3545, 95% confidence interval [CI] [−0.4272, −0.2818], *p* < 0.0001). The negative coefficient suggests that while holding other variables constant an increase in the IGF-1 Z-score is associated with a decrease in the expected value of the GAD 7 score. The exponentiated coefficient (0.7015) suggests that for each one-point increase in the IGF-1 *Z*-score the expected outcome of the GAD 7 score decreases by approximately 29.85% (95% CI [24.56%, 34.77%]).

### Patient Health Questionnaire-9 Generalized Linear Model

IGF-1 *Z-*score is the only statistically significant predictor of the PHQ-9 score (*β* = −0.1779, 95% CI [−0.2414, −0.1144], *p* < 0.0001). The negative coefficient suggests that while holding other variables constant, an increase in the IGF-1 *Z-*score is associated with a decrease in the expected value of the PHQ-9 score. The exponentiated coefficient (0.8370) suggests that for each one-point increase in the IGF-1 *Z-*score the expected outcome of the PHQ-9 score decreases by approximately 16.30% (95% CI [10.81%, 21.45%]).

### Rivermead Post-Concussion Symptoms Questionnaire Generalized Linear Model

IGF-1 *Z-*score is the only statistically significant predictor of the RPQ 13 score (*β* = −0.4980, 95% CI [−0.5616, −0.4344], *p* < 0.0001). The negative coefficient suggests that while holding other variables constant, an increase in the IGF-1 *Z-*score is associated with a decrease in the expected value of the RPQ 13 score. The exponentiated coefficient (0.6077) suggests that for each one-point increase in the IGF-1 *Z-*score the expected outcome of the RPQ 13 score decreases by approximately 39.23% (95% CI [35.23%, 42.97%]).

## Discussion

In this study, we analyzed serum IGF-1 levels, symptom severity, as well as depression and anxiety severity post-TBI. Pituitary labs were collected between 3 and 12 months following the injury, in accordance with the current consensus on the clinical evaluation timeline for hypopituitarism in this patient population.^22–23^ Approximately one-third of TBI sufferers experience persistent anterior pituitary dysfunction 12 months or more post-injury. The anterior pituitary hormones potentially affected include GH, adrenocorticotropic hormone, thyroid-stimulating hormone, luteinizing hormone, follicle-stimulating hormone, and prolactin.^24^ The population’s scores on the PHQ-9, GAD-7, and RPQ-13 closely align with those reported in other studies of participants with symptomatic TBI, highlighting the consistency and reliability of these instruments.^25^ Similarly, the average IGF-1 *Z-*score, which is slightly negative, aligns with findings from other studies assessing IGF-1 levels in the TBI population.^26^

Data showed significant negative correlations between IGF-1 levels and RPQ-13, GAD-7, and PHQ-9 scores in both mTBI and msTBI TBI. As IGF-1 *Z-*scores decreased below 0, there were significant increases in RPQ-13, GAD-7, and PHQ-9 scores, indicating a rise in TBI symptoms, depression, and anxiety, with most severe symptoms observed at *Z-*scores of negative 2 or below. This parallels trends observed in the literature, which demonstrate negative correlations between GH level and rates of disability, functional outcomes, and cognition in post-acute TBI.^2,4,27,28^ Maric et al. saw improved depression in chronic patients with TBI after receiving GH therapy.^29^ Similarly, a systematic review by Szarka et al. saw improvements in verbal and working memory, executive function, FIM scores, motor, and quality of life after GH administration, though results were mixed regarding rates of depression.^30^ Additionally, preclinical studies demonstrated a dose-dependent enhancement of hippocampal neurogenesis post-TBI in mice treated with IGF-1 as well as improved motor recovery and memory retention.^1^

GAD-7 and PHQ-9 scores for the mTBI group were moderately negatively correlated with IGF-1 *Z-*scores, indicating moderately correlated increase in symptoms of anxiety and depression with decreasing serum IGF-1. TBI symptom severity was strongly increased with decreasing serum IGF-1 levels for this group, as indicated by the strong negative correlation between RPQ-13 scores and IGF-1 *Z-*scores. PHQ-9 and GAD-9 scores strongly negatively correlated with IGF-1*Z-*scores in the msTBI group, suggesting a strong correlation between decreasing serum IGF-1 and increasing symptoms of depression and anxiety. Additionally, RPQ-13 scores were very strongly negatively correlated with IGF-1 *Z-*scores, indicating a significantly elevated TBI symptom severity with decreasing serum IGF-1.

Several interesting trends emerged in the analysis of participants with and without pre-morbid depression and anxiety. Firstly, while Kendall’s Tau correlations between IGF-1 and GAD-7 for all TBI groups were strongly to very strongly negative, it appears that a history of preinjury anxiety may predispose individuals with TBI and low IGF-1 to more severe anxiety post-injury. This is supported by the more strongly negative Kendall’s Tau correlations seen for the mTBI and msTBI groups with pre-morbid anxiety as compared with their counterparts without preinjury anxiety. Data also suggest that anxiety experienced may generally increase with TBI severity and decreasing IGF-1 level, which may inform neuropsychological screening, monitoring, and treatment. Irrespective of a history of depression, PHQ-9 scores increased across all TBI groups as IGF-1 *Z-*scores decreased, suggesting that following a TBI, the severity of depression symptoms is more closely associated with IGF-1 levels than with a previous history of depression.

The GLMs indicate that the IGF-1 *Z-*score is a significant predictor for all three measures: GAD-7, PHQ-9, and RPQ-13. Specifically, a one-point increase in the IGF-1 *Z-*score is associated with a 29.85% decrease in anxiety symptoms (GAD-7), a 16.30% reduction in depression severity (PHQ-9), and a 39.23% decrease in post-TBI symptom severity (RPQ-13). These findings suggest that elevated IGF-1 levels may have protective effects against anxiety, depression, and post-TBI symptoms.

Our findings suggest that decreased circulating IGF-1 is associated with increased RPQ-13, GAD-7, and PHQ-9 scores in both mTBI and msTBI, reflecting increased post-injury symptom severity and higher rates of depression and anxiety.

### Limitations

This study investigated the relationship between IGF-1 levels and TBI symptom severity using standardized assessment tools. However, the retrospective observational design limits the ability to establish causality, and reliance on electronic medical record data may introduce bias. Additionally, the study included participants who had persistent symptoms following TBI or continued to follow up in the clinic meeting criteria for IGF-1 level monitoring, which may limit the generalizability of the findings to individuals with varying symptom trajectories. Furthermore, the exclusion of patients outside the 18–65 age range restricts the generalizability of the findings to broader age demographics. The study also did not control for potential confounding factors such as pre-existing conditions and medications that could influence IGF-1 levels and psychological outcomes. The use of self-reported questionnaires introduces an element of subjectivity and potential bias. Future research should employ a prospective design, include a broader age range, and account for additional confounding variables to better elucidate the causal pathways linking IGF-1 to TBI outcomes.

## Conclusion

This study reveals the significant inverse relationship between serum IGF-1 levels and the severity of post-injury symptoms, depression, and anxiety in patients with both mTBI and msTBI. The negative correlations between IGF-1 *Z-*scores and the RPQ-13, GAD-7, and PHQ-9 scores suggest that IGF-1 may play a role in modulating neuropsychological outcomes following TBI. Our data indicate that decreased IGF-1 levels are associated with increased symptom severity and heightened rates of depression and anxiety, highlighting IGF-1 as a potential biomarker for post-TBI recovery. These findings underscore the potential for IGF-1 to inform therapeutic strategies aimed at mitigating sequelae in patients with TBI. To further elucidate the causal mechanisms and therapeutic potential of IGF-1 in TBI, a prospective study is needed to provide deeper insights into its neuroprotective roles and inform novel interventions to enhance recovery and quality of life for TBI survivors.

## Transparency, Rigor, and Reproducibility Summary

This retrospective observational study was not preregistered due to its nature, which involved analyzing existing data collected from September 1, 2021, to September 1, 2023, at an Academic Brain Injury Center. The analysis plan was not formally preregistered, as the study was conducted using data available in electronic medical records. The sample size was determined based on the availability of eligible participants who met the inclusion criteria, resulting in 138 individuals in the mild TBI (mTBI) group and 102 in the moderate-to-severe TBI (msTBI) group. All participants included had complete RPQ-13, GAD-7, and PHQ-9 responses, and IGF-1 values available, as outlined in the study protocol. Blinding was not applicable in this retrospective study since it involved the analysis of pre-existing data. Biofluid samples for IGF-1 analysis were collected as part of routine clinical care by Quest Diagnostics, processed according to their standard protocols, and stored under conditions ensuring data integrity. All analytical reagents and equipment used for IGF-1 measurement are widely available from commercial sources, ensuring the reproducibility of the findings. The primary clinical outcome measures, including RPQ-13, GAD-7, and PHQ-9 scores, are established standards in the field for assessing post-TBI symptoms, anxiety, and depression. Statistical analyses were conducted using SAS Enterprise Guide 8.3, with Kendall’s Tau correlations employed to assess relationships due to their robustness in handling noncontinuous data. Multiple comparisons were managed using a significance level of *α* = 0.05, with adjustments for multiple testing as necessary. No internal or external validation studies are currently planned, nor is there ongoing replication of the findings. The purpose of this study is to inform planning for a prospective study evaluating IGF-1 as a biomarker for symptoms severity, which is planned pending funding. Data from this study are not available in a public archive. However, data requests can be directed to the corresponding author, subject to IRB guidelines. There is no analytic code associated with this study available for public access. Biofluid samples used in this study were obtained as part of routine clinical assessments and are not available for future research due to limited quantities and institutional policies. This publication will be available in an Open Access format, ensuring public accessibility upon publication.

## Abbreviations Used

GH: growth hormone
GHD: growth hormone deficiency
TBI: Traumatic brain injury
