# Supplementary material for: IGF-1 as a Biomarker for Symptom Severity in Adult Traumatic Brain Injury: Evidence from an Observational Study
Source: Neurotrauma Rep. 2025 Apr 21;6(1):345–54. doi: 10.1089/neur.2025.0009 (PMC12281112; doi:10.1089/neur.2025.0009)
Supplement: Supplementary Table S1 [file neur.2025.0009_supplementary_table_s1.docx]

**Supplemental table 1.** Interpretation of Kendall’s Tau correlations.

| **Strength** | **Kendall** |
| --- | --- |
| Negligible | 0.00 |
| Weak | 0.06 |
| Moderate | 0.26 |
| Strong | 0.49 |
| Very Strong | 0.71 |
